# Application of PVA hydrogel loaded with luteolin nanoparticles in anti EMT treatment after GBM

**DOI:** 10.1016/j.mtbio.2025.101956

**Published:** 2025-06-07

**Authors:** Long Zhou, Qingyu Zhao, Lijuan Gu, Renfu Tian, Yong Li, Xiaoxing Xiong

**Affiliations:** a Department of Neurosurgery, Renmin Hospital of Wuhan University, Wuhan, 430060, Hubei, China; b Central Laboratory, Renmin Hospital of Wuhan University, Wuhan, China; c Department of Neurosurgery, The Central Hospital of Enshi Tujia and Miao Autonomous Prefecture, Enshi City, Hubei Province, China; d Department of Neurosurgery, The Second Affiliated Hospital of Zhejiang University School of Medicine, Hangzhou, China

**Keywords:** Luteolin, GBM, Proliferation, EMT, β-catenin

## Abstract

**Background:**

Glioblastoma multiforme (GBM) is the most common and worst-prognosed primary malignant tumor in the central nervous system. Epithelial mesenchymal transition (EMT) is an important cause of postoperative invasion and recurrence in GBM. Due to the presence of the blood-brain barrier, local therapy may be the preferred route for treatment of GBM after surgery.

**Methods:**

Our objective is to develop an anti- EMT functional hydrogel for post-GBM surgery tamponade. We fabricated self-assembled luteolin nanoparticles (LU NPs) via the solvent evaporation method, and characterized their morphology and particle size using scanning electron microscopy (SEM), transmission electron microscopy (TEM), and dynamic light scattering (DLS). Furthermore, biological evaluations were conducted through CCK-8 assays, EdU staining, flow cytometry for cell cycle analysis, scratch assays, transwell assays and western blot. Ultimately, we constructed a polyvinyl alcohol (PVA) hydrogel loaded with luteolin nanoparticles and validated its therapeutic efficacy in vivo experiments.

**Results:**

We successfully synthesized LU NPs, which significantly inhibited GBM cell proliferation, as well as GBM cell invasion and migration in vitro. Furthermore, LU NPs downregulated the EMT signaling pathway. We discovered that the observed anti-tumor effects of LU NPs were dependent on the function of β-catenin. Additionally, we successfully constructed a PVA hydrogel loaded with LU NPs (LU@gel). Finally, in the postoperative model of intracranial GBM xenograft in mice, LU@gel effectively suppressed GBM proliferation and EMT, significantly prolonging the survival time of the mice.

**Conclusions:**

In summary, we have demonstrated that LU@gel exhibits potent anti-GBM effects, primarily attributed to the inhibition of β-catenin-mediated cell proliferation and EMT.

## Graphical abstract

We have developed a synthesis method for Luteolin nanoparticles and encapsulated them within a PVA hydrogel for in situ packing therapy following GBM resection. We discovered that Luteolin nanoparticles can inhibit the cell cycle and EMT through the β-catenin pathway, exhibiting therapeutic effects on GBM.

**Figure:**
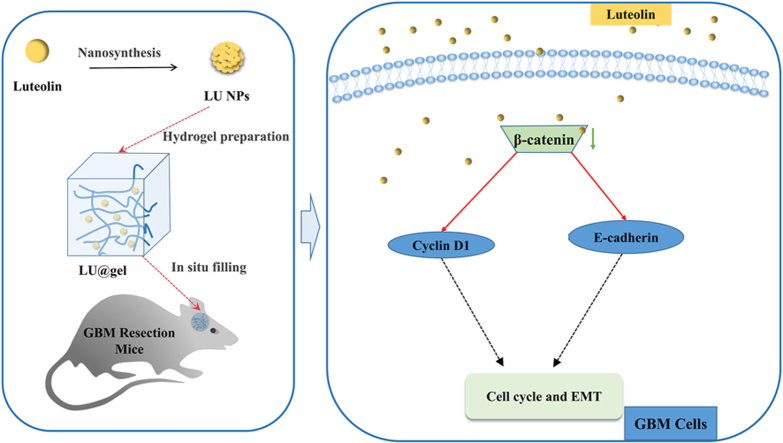

## 1 Introduction

Glioblastoma multiforme (GBM) is the most common and worst-prognosed primary malignant tumor in the central nervous system [1]. The median survival time of GBM patients is only 14–15 months, characterized by poor clinical prognosis, high invasiveness, high mortality, and easy recurrence [2]. Standard treatment methods for GBM include surgical resection, local radiotherapy, and adjunctive chemotherapy [3]. However, surgical removal of the tumor does not mean the complete elimination of glioma cells [[4], [5], [6]]. Epithelial-mesenchymal transition (EMT) is an important process where epithelial cells lose cell polarity and adhesiveness, thus transforming into mesenchymal cells, resulting in increased invasive or metastatic phenotypes [7]. This high invasion and migration ability caused by EMT ultimately leads to tumor recurrence [8]. Due to the delicate anatomical structure of the brain and the surrounding blood-brain barrier (BBB), traditional drug injection rarely achieves successful therapeutic effects [[9], [10], [11]]. Therefore, finding an effective treatment method to passing through the BBB and inhibit EMT after GBM surgery has become a hotspot in current anti-GBM research.

EMT is the process in which epithelial cells transform into mesenchymal cells. EMT plays a crucial role in the progression, distant metastasis, and treatment resistance of various malignant tumors [12]. When epithelial cells are stimulated by various factors (growth factor signaling, tumor-stromal cell interaction, hypoxia, etc.), they can activate this program to acquire mesenchymal cell characteristics (including motility, invasiveness, and anti-apoptosis), thus losing their differentiation characteristics (including cell-cell adhesion, planar and apical polarity, and lack of motility). This process usually starts with the loss of epithelial cell polarity and the breakdown of cadherin-related cell-cell adhesion. These results provide a basis for further elucidating the invasion and distant metastasis of tumor cells [13]. Characteristic changes of EMT mainly include the downregulation of epithelial markers (E-cadherin) and the upregulation of mesenchymal markers (vimentin and N-cadherin). Current research shows that EMT plays a central role in the invasion, aggressiveness, and recurrence of GBM [14]. EMT is regulated by multiple signaling pathways, such as TGF β, Wnt/β-catenin, Hedgehog, and Notch signaling pathways, which trigger EMT by stimulating transcription factors such as Snail, Twist, and ZEB1/2 [[15], [16], [17], [18]]. Among all signaling pathways, the β-catenin pathway plays a crucial role in the regulation of EMT [16]. In various cancer cells, β-catenin accumulates extensively in the cytoplasm and subsequently translocates into the nucleus, where it forms a complex with transcription factors such as T-cell factor/Lymphoid enhancing factor (TCF/LEF). This complex activates downstream target genes (such as c-myc and cyclinD1), promoting cell cycle progression or producing abnormal proteins, which in turn induce epithelial-mesenchymal transition (EMT) [[19], [20], [21], [22]].

Luteolin (LU) belongs to the flavonoid polyphenol family, originally extracted from plants such as thyme and sage [[23], [24], [25]]. Studies have shown that luteolin has various biological and pharmacological activities, such as antibacterial, antiviral, antioxidant, anti-tumor, and immunomodulatory effects [[26], [27], [28], [29], [30], [31], [32]]. Luteolin exhibits anti-cancer effects in various types of cancer by inducing cell death, slowing cell proliferation, and preventing cell growth. In gliomas, Wang et al. pointed out that luteolin inhibits glioma cell proliferation through endoplasmic reticulum stress and mitochondrial pathways [33]. Anson et al. mentioned that luteolin can reduce cell proliferation by inhibiting the epidermal growth factor signaling pathway in gliomas [34]. Recent studies have shown that luteolin can inhibit the EMT process in tumor cells through multiple pathways, making it a potential therapeutic drug for inhibiting EMT after glioma surgery [[35], [36], [37]]. However, like most natural bioactive molecules, luteolin faces issues such as low water solubility, low bioavailability, low tissue targeting, and low blood-brain barrier (BBB) penetration rate, which greatly limit its in vivo application [38]. Therefore, developing an effective drug delivery system to address these in vivo application challenges is a crucial step in promoting the clinical translation of luteolin. In recent studies, we have successfully constructed various nanoparticle drug delivery systems through the self-assembly of small molecules and applied them in the treatment of neuro-oncology and stroke [[39], [40], [41], [42]]. In this experiment, we constructed self-assembled luteolin nanoparticles (LU NPs) to address its issue of poor water solubility.

Due to the presence of the BBB, local therapy may be the preferred route for delivering drugs to treat GBM. Hydrogel is one of the most commonly used local drug delivery substrates [43]. Hydrogels are usually formed by a three-dimensional network structure of macromolecules and can be used as drug carriers, cell delivery carriers, and tissue engineering materials. Hydrogels have adjustable biological, chemical, physical, and mechanical properties that match soft/hard tissues, can cover dead angles of lumen, simulate many characteristics of natural cells and tissue environments, and have many unique advantages when used as local therapeutic agents in anti-tumor applications [44]. Polyvinyl alcohol (PVA) is a synthetic macromolecular polymer with relatively excellent mechanical properties, biocompatibility, low cost, and stability [45]. PVA-based hydrogels are attractive polymeric materials that are increasingly being used in disease treatment, physiological signal monitoring, and other aspects, with tremendous biomedical application potential. This study aims to encapsulate luteolin nanoparticles within PVA hydrogels to construct LU@gel for in-situ treatment following glioma surgery.

We found that in the GBM orthotopic tumor model, LU@gel slowed the growth of GBM and significantly inhibited the EMT process of GBM. In summary, our research results indicate that LU@gel may be a promising drug for inhibiting GBM recurrence after surgery.

## 2 Materials and methods

### 2.1 Antibody and reagents

Luteolin and Polyvinyl alcohol (PVA) (1799) was purchased from Shanghai Aladdin Biochemical Technology Co., Ltd. (Shanghai, China). PVA (1789) was purchased from Sigma-Aldrich LLC [USA]. The antibodies used for immunostaining were anti-mouse-Cyclin D1 antibody (60186-1-Ig, Proteintech); anti-rabbit-E-cadherin antibody (20874-1-AP; Proteintech); anti-rabbit-N-cadherin antibody (22018-1-AP; Proteintech); anti-mouse-Bcl-2 (68103-1-Ig, Proteintech), anti-rabbit-Vimentin antibody (5741; CST); anti-rabbit-β-catenin antibody (8480; CST); anti-rabbit-GAPDH antibody (GB11002-100; Servicebio) and anti-rabbit-Tubulin antibody (GB11017-100; Servicebio).

### 2.2 Preparation and characterization of LU NPs and LU@gel

LU NPs were prepared using a standard emulsion process. First, 10 mg of Luteolin was dissolved in 1 ml of an ethyl acetate/methanol mixture (9:1 ratio). This solution was then added dropwise, while vortexing, to a 50 ml tube containing 3 ml of polyvinyl alcohol (PVA) solution (2.5 wt%, molecular weight 30,000–70,000 Da). Next, the emulsion was sonicated on ice for a total of 100 s, in intervals of 10 s with 5-s breaks. The resulting mixture was poured into a pre-cooled beaker containing 30 ml of PVA solution (0.3 wt%, 1789, molecular weight 30,000–70,000 Da) under vigorous stirring. The mixture was stirred overnight at 4 °C. Following this, the nanoparticles were collected by centrifugation at 18,000 rpm for 30 min at 4 °C. The nanoparticles were washed three times with 30 ml of water, each time collected by centrifugation (18,000 rpm at 10 °C for 30 min). Finally, the nanoparticles were resuspended in 1 ml of distilled water and stored. To prepare LU@gel, the LU NPs (10 mg/ml) were mixed with PVA (1799) to achieve a final PVA concentration of 8 %, then subjected to three freeze-thaw cycles in a −80 °C freezer.

### 2.3 Dynamic light scattering (DLS)

LU NPs were diluted to a 1 mg/mL aqueous solution. The hydration diameter and zeta potential were measured using a Dynamic Light Scattering device (Zetasizer Nano ZSP, Malvern Instruments Ltd., UK).

### 2.4 Transmission electron microscope (TEM)

LU NPs suspensions are deposited onto porous carbon-coated copper grids (SPI, West Chester, PA, USA) and allowed to adsorb for a few minutes. The grids were left at fume hood until completely dried. The prepared grids are then placed in a transmission electron microscope (Fei TECNAI tf20 TEM, Thermo Fisher Scientific, Waltham, Ma, USA), where high-resolution images are captured to examine the nanoparticles' morphology, size, and distribution.

### 2.5 Scanning electron microscopy (SEM)

Gold coating was performed on the samples using a sputter coater under vacuum and argon atmosphere at a sputtering current of 40 mA for 60 s (auto fine coater JFC-1200, JEOL Ltd., Japan). SEM was performed using XL-30 scanning electron microscopy (Fei, Hillsboro, or, USA) at an acceleration voltage of 5 kV and a magnification of 20,000 times.

### 2.6 Atomic force microscopy (AFM)

The LU NPs were analyzed using a scanning probe microscope (SPM, Dimension ICON, Bruker, Malaysia). Thin films of LU NPs were prepared by drop-casting their dispersion onto a clean 1 cm^2^ glass slide, followed by drying under ambient conditions. Measurements were carried out in tapping mode at room temperature (25 °C) using a silicon tip with a resonance frequency of approximately 300 kHz and a force constant of 40 N/m. The images, captured at a resolution of 512 × 512 pixels over a scan area of 2 × 2 μm^2^, were processed using NanoScope Analysis software to determine particle size and surface morphology.

### 2.7 X-ray photoelectron spectroscopy (XPS)

XPS (ESCALAB Xi^+^, Kratos Analytical Ltd., UK) endowed with an Al Kα source (hv = 1486.71 eV) was used to analyze the material's chemistry. A survey spectrum with an energy range of 0–1350 eV was obtained.

### 2.8 Rheological testing

Rheology was performed on an Anton-paar MCR 702e Rheometer (Anton-paar, Austria) using oscillatory mode with a plane/plane geometry and a gap of 0.1 mm, with the plate rotor (P35/Ti/SB) and sample hood each having a diameter of 35 mm. Frequency sweep experiments were performed with frequency from 0.1 Hz to 10 Hz. The storage modulus (G′) and the loss modulus (G″) were extracted.

### 2.9 Cell culture

The mouse GBM cell lines GL261 and 293T originally from ATCC were cultured at 37 °C and 5 % CO2, in high-glucose DMEM Medium (Gibco, USA) supplemented with 10 % fetal bovine serum (Gibco, USA), 100 U/mL penicillin, and 100 μg/mL streptomycin.

### 2.10 Cell viability assay

The anti-proliferative effects of luteolin and luteolin nanoparticles on GL261 cells were evaluated using the Cell Counting Kit-8 (CCK8) assay, as per the protocol provided by Beyotime Inc. Initially, GL261 cells were seeded into 96-well plates at a density of 10,000 cells per well and allowed to adhere for 24 h. After the attachment period, the medium was replaced with DMEM containing of either luteolin or luteolin nanoparticles with the concentration of 0, 1, 2, 4, 8, 16 and 32 μg/ml. After treatment of 24 h, 10 μL of CCK8 solution was added to each well and incubated for 1 h at 37 °C. The absorbance was then measured at 450 nm using a microplate reader to assess cell viability.

### 2.11 EdU DNA synthesis analysis

The Cell Light EdU Apollo 567 In Vitro Culture Kit (RiboBio, Guangzhou, China) was used to assess cell growth. Initially, 10,000 GL261 cells were plated in 96-well plates with 100 μL of DMEM. The cells were treated with LU NPs at concentrations of 0, 5, 10, and 20 μg/ml for 24 h. Following treatment, the cells were incubated with 50 μL of EdU medium for 2 h, then fixed with 4 % paraformaldehyde for 30 min. Next, 100 μL of 1x Apollo® cocktail was added and incubated for 30 min. Afterward, the cells were counterstained with 1x Hoechst 33342 in the dark for 30 min. Fluorescence images of Hoechst 33342 and EdU staining were captured using a fluorescence microscope (BX51, Olympus, Japan) to observe and analyze cell growth.

### 2.12 Cell migration and invasion

Cell migration was evaluated using a wound healing assay. GL261 cells were plated in a 6-well plate at a density of 5 × 10^5^ cells per well and incubated overnight to allow for cell attachment. A scratch was made across the cell monolayer with a 200-μL pipette tip, and the cells were then washed three times with PBS. The cells were cultured in serum-free medium, and images were taken at designated time points to observe wound closure. The extent of migration was measured using ImageJ. Cell invasion was assessed with a transwell assay. GL261 cells in serum-free medium were placed in the upper chamber of a Transwell insert (24-well format, 8 μm pore size, Corning) pre-coated with matrigel. The lower chamber contained medium with 10 % serum to attract the cells. After 24 h of incubation, cells that had invaded through the matrigel and membrane were stained with crystal violet, photographed under a microscope, and counted to determine their invasive potential.

### 2.13 Western blot analysis

GL261 cells were lysed on ice using a modified RIPA buffer (No. p0013b, Beyotime Biotechnology, China) for approximately 30 min. The lysates were then centrifuged at 12,000 rpm for 15 min. The protein concentration of the samples was measured using the BCA protein assay. Following this, the lysates were heated at 100 °C for 5 min, then mixed with loading buffer. Equal amounts of protein were loaded onto an 8–12 % SDS-PAGE gel and separated. The proteins were then transferred to a nitrocellulose membrane. The membrane was blocked with 5 % skimmed milk for 1 h, followed by incubation with primary and secondary antibodies at 4 °C for 1 h each.

### 2.14 Immunofluorescence stained

Cells were fixed with 4 % paraformaldehyde for 30 min and then permeabilized with 0.2 % Triton X-100 in PBS at room temperature for 15 min. The slides were subsequently washed three times with PBS. Next, the slides were treated with 1 % BSA at room temperature for 30 min to block non-specific binding. After blocking, a sufficiently diluted primary antibody was applied to the slides, which were then placed in a humidified chamber and incubated overnight at 4 °C. Following primary antibody incubation, the slides were exposed to a secondary antibody (Antgene, Wuhan, China) in a dark, humid environment at 37 °C for 1 h. After incubation, the slides were mounted with DAPI to counterstain the nuclei and reduce fluorescence quenching. Finally, the slides were examined under a fluorescence microscope (Olympus BX51, Japan) to capture the images.

### 2.15 Construction of GL261-luc Cell line

To develop the GL261-luc cell line, we began by preparing lentiviral vectors that encode luciferase. These vectors were then used to transfect 293T cells, along with packaging plasmids, to produce lentivirus. After 48 h, the lentivirus was harvested and concentrated. Next, GL261 cells were plated in a 6-well plate and transduced with the concentrated viral solution, which included polybrene, for 24–48 h. Post-transduction, we selected for stable integration of the virus using puromycin and expanded the resulting cells. To confirm successful transduction, we performed a luciferase assay and, if necessary, fluorescence microscopy. Finally, the GL261-luc cells were cryopreserved for future use.

### 2.16 Intracranial xenograft model

Male C57BL/6J mice, aged 6–8 weeks, were housed in a sterile environment for the study, which was approved by the Animal Protection and Utilization Committee of the Renmin Hospital of Wuhan University. GL261-luc cells, harvested during their logarithmic growth phase, were resuspended in PBS to a concentration of 1 × 10^5^ cells/μL. Following anesthesia with isoflurane, a 1–2 mm craniotomy was performed over the right frontal lobe of the mouse. Using stereotaxic techniques, 1 × 10^5^ GL261 cells were injected into the right ventricle of each mouse. Post-surgery, the mice were given appropriate care, including antibiotic treatment and close monitoring for any signs of distress. Tumor progression was monitored through regular observations and luciferase bioluminescence imaging system detection.

### 2.17 Determining the efficacy of GBM treatment

Seven days post-implantation, the mice were divided into four groups: control, TMZ, blank gel, and LU@gel. In the control and TMZ groups, PBS and TMZ were administered via the caudal vein every two days for a total of six injections. For the blank gel and LU@gel groups, 5 μL of either blank gel or LU@gel were applied directly to the tumor site immediately following surgery. To evaluate treatment efficacy, mice were monitored regularly and euthanized upon exhibiting severe neurological symptoms or significant weight loss (greater than 20 % of their body weight). The brains were then extracted, weighed, and fixed in 4 % paraformaldehyde. The fixed brains were preserved for further analysis and subsequently embedded in paraffin.

### 2.18 Statistical analysis

All data were collected in triplicate and reported as mean and standard deviation. All experimental results were presented as mean ± standard deviation (SD). Student's t-test was used to analyze the differences between two groups. A one-way analysis of variance (ANOVA) was used for comparison among multiple groups, followed by a Tukey post hoc test. The Shapiro-Wilk test was employed to assess normality, and the Mann-Whitney *U* test was used to evaluate data with non-normal distributions. A p value of less than 0.05 was considered as statistical significance. All statistical graphs were created using GraphPad Prism 8.0.

## 3 Result

### 3.1 Synthesis and Characterization of LU NPs

**Fig. 1:**
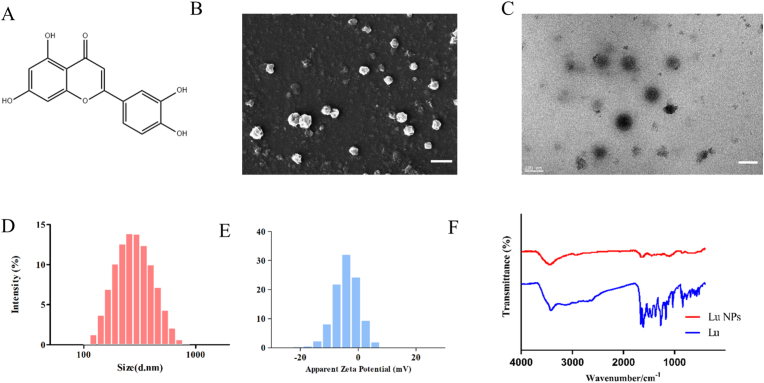
**Synthesis and Characterization of LU NPs.** (A) Molecular structure of luteolin. (B) Representative SEM image of LU NPs. Scale bar: 500 nm. (C) Representative TEM image of LU NPs. Scale bar: 100 nm. (D) Mean sizes of LU NPs detected by DLS. (E) Mean zeta potential of LU NPs detected by DLS. (F) FTIR of free LU and LU NPs.

In recent years, natural flavonoid compounds have garnered increased interest due to their impressive biological activities. Among these, luteolin, a type of flavone, is particularly noted for its potent anti-inflammatory and anti-tumor effects (Fig. 1A). However, its use in treating central nervous system tumors is limited by its poor water solubility and insufficient ability to cross the blood-brain barrier. To address these limitations, we prepared LU NPs using a solvent evaporation method to enhance both water solubility and blood-brain barrier permeability. Scanning electron microscopy (SEM) and transmission electron microscopy (TEM) revealed that LU NPs were solid, spherical particles with an approximate diameter of 200 nm (Fig. 1B and C). Our further investigate the three-dimensional structure of LU NPs, AFM analysis revealed that LU NPs exhibit a spherical morphology but are coated with a membrane-like structure on their surface. This may be attributed to the presence of residual PVA in the dispersant, which is difficult to remove (Fig. S1). Dynamic light scattering (DLS) measurements indicated an average particle size of 187 nm and a zeta potential of −14.8 mV (Fig. 1D and E). Fourier transform infrared spectroscopy demonstrated significant changes in the absorption peaks of LU NPs compared to free luteolin, confirming the successful synthesis of the nanoparticles (Fig. 1F). After repeated washing and vacuum freeze-drying of LU NPs, we observed that a substantial amount of PVA remained within the particles. Based on the morphology of LU NPs, we hypothesize that the formation mechanism of LU NPs likely involves the extensive formation of hydrogen bonds between luteolin and hydroxyl groups located on the side chains of PVA. These hydrogen bonds facilitate the self-assembly and entanglement of PVA long chains, ultimately leading to the formation of spherical nanoparticles.

### 3.2 LU NPs inhibit GBM cell proliferation via the β-catenin/Cyclin D1 pathway

**Fig. 2:**
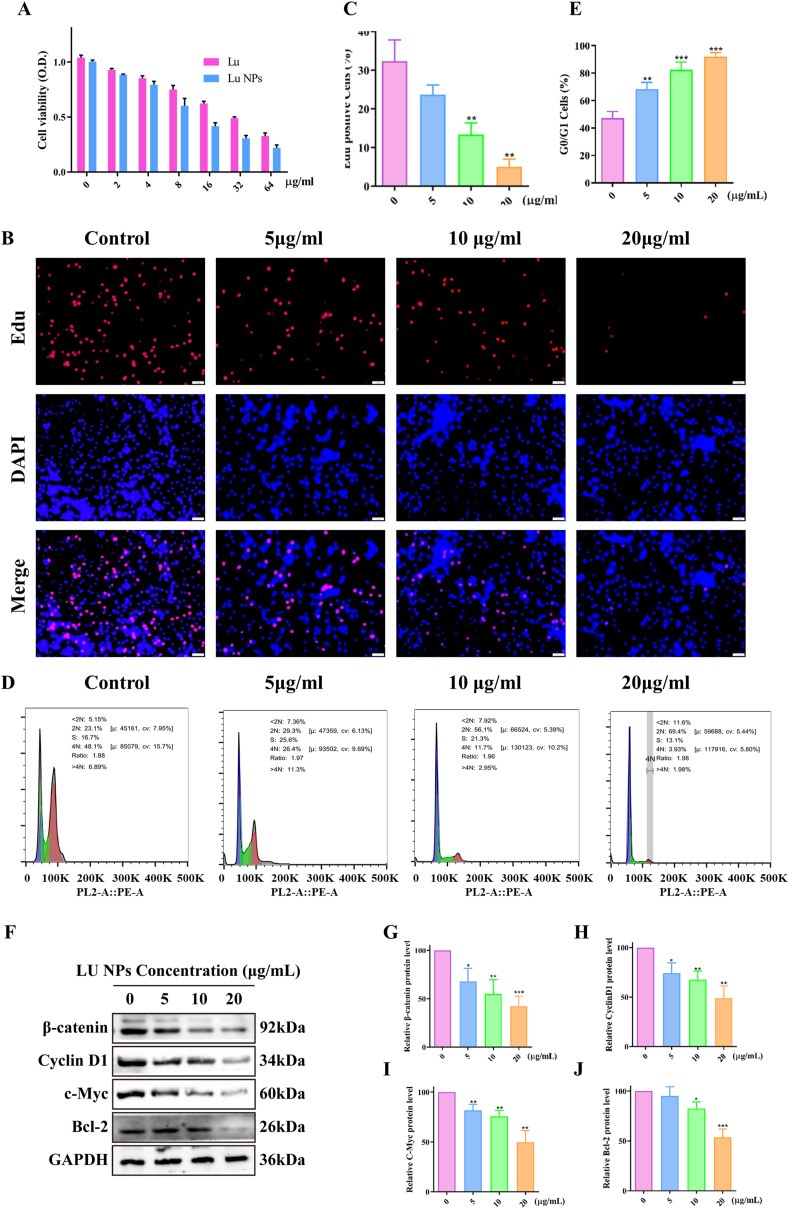
**LU NPs Inhibit GBM Cell Proliferation via the β-catenin/Cyclin D1 Pathway. (**A) Cell viability measured by CCK8 assay after LU NPs treatment with various concentrations (0, 2,4,8,16,32, 64 μg/mL). (B, C) EdU assay shown that LU NPs inhibited DNA synthesis in GL261 cells. Scale bar: 20 μm. (n = 3) ∗∗P < 0.01. (D, E) Cell cycle analysis by flow cytometry of GL261 cells after been treated with various concentrations (0, 5, 10, 20 μg/mL) LU NPs. (n = 3) ∗∗P < 0.01, ∗∗∗P < 0.001. (F–J) Cell cycle related protein expression quantified with western blot. (n = 3) ∗P < 0.05, ∗∗P < 0.01, ∗∗∗P < 0.001.

We evaluated the anti-proliferative effects of LU NPs on GBM cells using the CCK-8 assay. The results demonstrated that LU NPs inhibited GL261 cell proliferation in a dose-dependent manner, with an IC_50_ value of 16.9 μg/ml (Fig. 2A, S2). To further assess the anti-proliferative effects, we performed an EdU DNA assay, which showed a significant reduction in the percentage of EdU-positive cells in the presence of LU NPs (Fig. 2B and C). We have further investigated the impact of LU NPs on the cell cycle progression. Flow cytometry analysis revealed that after 24 h of treatment with LU NPs, the proportion of GL261 cells residing in the G0/G1 phase increased in a dose-dependent manner (Fig. 2D and E). Furthermore, Western blot analysis demonstrated a corresponding dose-dependent decrease in β-catenin protein levels, accompanied by a dose-dependent reduction in the level of the cell cycle-related protein Cyclin D1, C-Myc and Bcl-2 (Fig. 2F–J). These findings collectively indicate that LU NPs inhibit glioblastoma by reducing cell proliferation in GL261 cells.

### 3.3 LU NPs inhibit GBM cell EMT via the β-catenin/EMT pathway

**Fig. 3:**
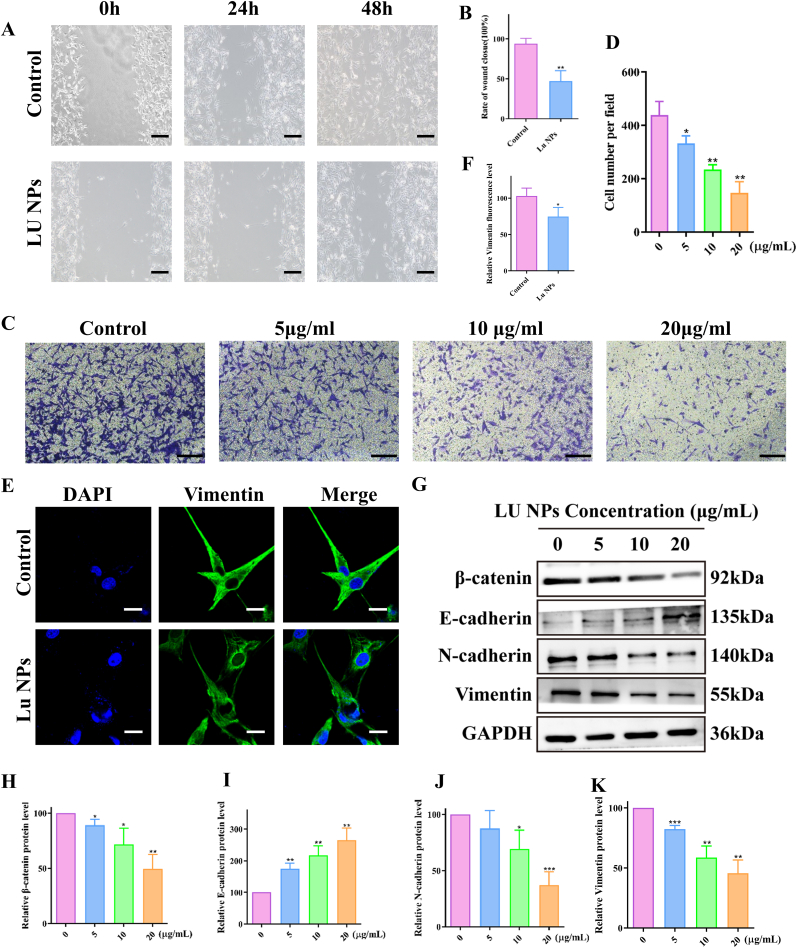
**LU NPs Inhibit GBM Cell Epithelial-Mesenchymal Transition (EMT) via the β-catenin/EMT Pathway.** (A, B) Wound healing in scratch assays demonstrated that LU NPs suppressed the migration of GL261 cells. Scale bar: 20 μm. (n = 3) ∗∗P < 0.01. (C, D) Transwell assay was performed to detect the migration and invasion in GBM cells. Scale bar: 20 μm. (n = 3) ∗P < 0.05, ∗∗P < 0.01. (E, F) Representative fluorescent images of Vimetin in GL261 cells. Scale bar: 10 μm. (n = 3) ∗P < 0.05. (G–K) EMT related protein expression quantified with western blot. (n = 3) ∗P < 0.05, ∗∗P < 0.01, ∗∗∗P < 0.001.

We investigated the effect of LU NPs on epithelial-mesenchymal transition (EMT) in GBM cells. Both scratch assays (Fig. 3A and B) and transwell assays (Fig. 3C and D) revealed that LU NPs significantly reduced the invasion and migration of GL261 cells. Immunofluorescence microscopy showed markedly lower fluorescence levels of Vimentin in LU NPs-treated cells compared to controls (Fig. 3E and F). To further explore the impact of LU NPs on the EMT pathway, we performed immunoblotting to assess changes in β-catenin and EMT markers following LU NPs treatment. The analysis showed a dose-dependent decrease in N-cadherin, Vimentin, andβ-catenin, alongside a dose-dependent increase in E-cadherin (Fig. 3G–K). These results indicate that LU NPs inhibit GBM cell EMT by targeting the β-catenin/EMT signaling pathway.

### 3.4 Elevating β-Catenin Activity Rescued LU NPs Inhibition‐Mediated GBM Cell proliferation and EMT

**Fig. 4:**
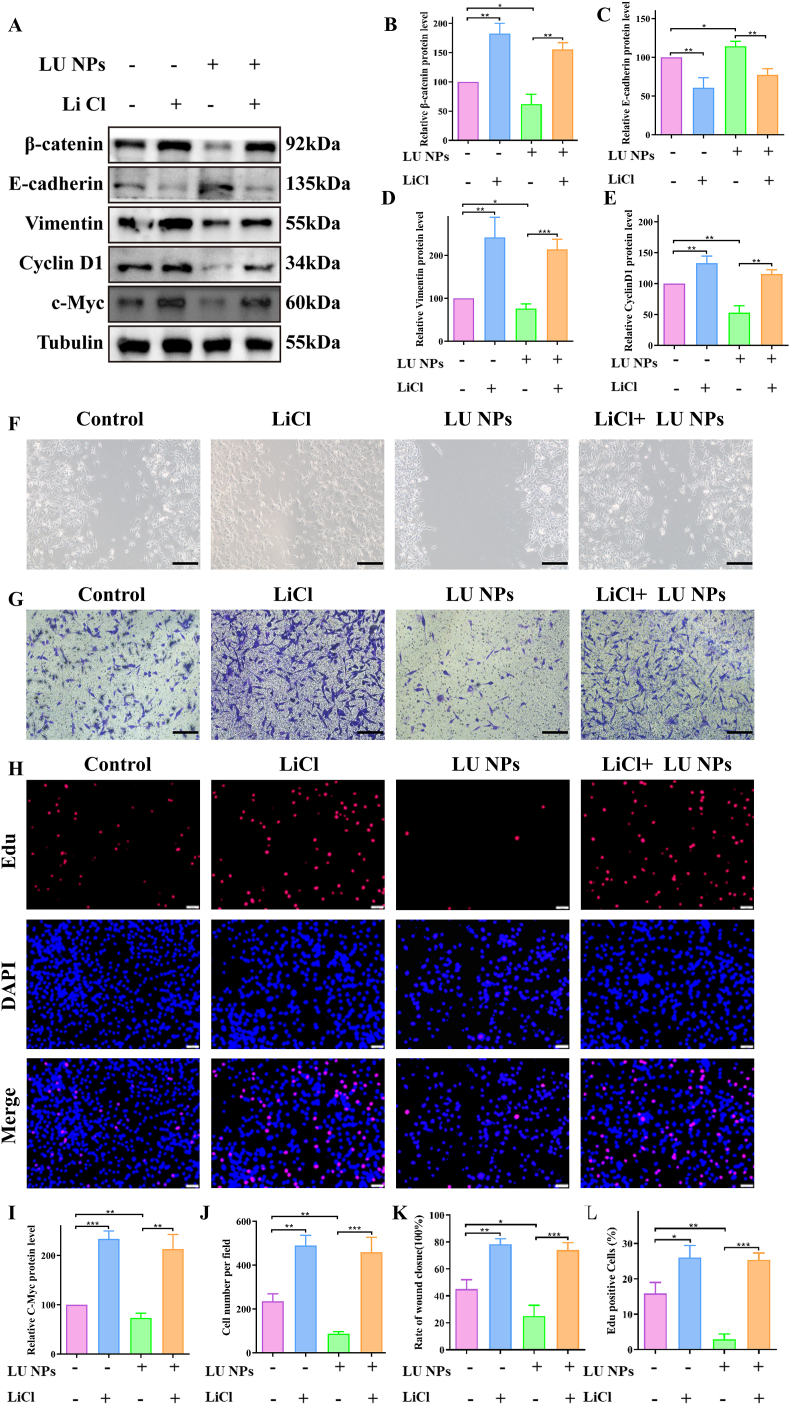
**Elevating β-Catenin Activity Rescued LU NPs Inhibition‐Mediated GBM Cell Proliferation and EMT.** (A–E, I) EMT related protein expression after LiCl processing quantified with western blot. (n = 3) ∗P < 0.05, ∗∗P < 0.01, ∗∗∗P < 0.001. (F, J) Scratch assays after LiCl processing of GL261 cells. Scale bar: 20 μm. (n = 3) ∗∗P < 0.01, ∗∗∗P < 0.001. (G, K) Transwell assay was performed to detect the migration and invasion after LiCl processing in GBM cells. Scale bar: 20 μm. (n = 3) ∗P < 0.05, ∗∗P < 0.01, ∗∗∗P < 0.001. (H, L) EdU assay after LiCl processing in GL261 cells. Scale bar: 20 μm. (n = 3) ∗P < 0.05, ∗∗P < 0.01, ∗∗∗P < 0.001.

To further substantiate the antitumor effect of LU NPs mediated through the β-catenin pathway, LiCl, an agonist of β-catenin known to enhance β-catenin signaling, was added to the cells 12 h post-treatment with LU NPs. Western blot analysis was employed to verify the relevant proteins involved in epithelial-mesenchymal transition (EMT) and cell cycle pathways. The results demonstrated that the downregulatory effects of LU NPs on Vimentin, Cyclin D1, and C-Myc were reversed by LiCl, while the upregulation of E-cadherin by LU NPs was also attenuated by LiCl (Fig. 4A–E, 4I). Scratch wound healing assays (Fig. 4F and J) and transwell assays (Fig. 4G and K) indicated that the inhibitory effects of LU NPs on migration and invasion were partially mitigated when β-catenin signaling was augmented by LiCl in GL261 cells. Furthermore, EdU assays revealed that the suppressed proliferation activity of GL261 cells by LU NPs was restored upon the elevation of β-catenin signaling by LiCl (Fig. 4H and L). Collectively, these findings suggest that LU NPs inhibit the proliferation and EMT of GBM cells by suppressing the activation of β-catenin signaling.

### 3.5 Preparation and characterization of LU@gel

**Fig. 5:**
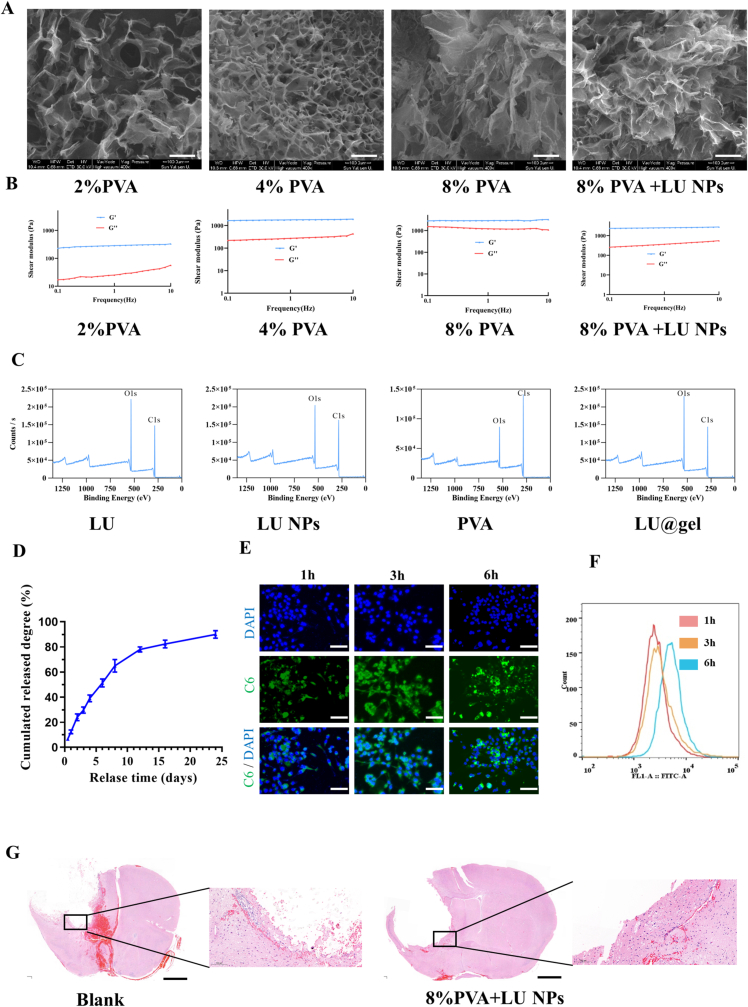
**Preparation and Characterization of LU@gel.** (A) Representative SEM image of LU@gel. Scale bar: 100 μm. (B) Rheological testing of LU@gel. (C)XPS testing of LU@gel. (D) Release curve of Luteolin released from LU@gel. (E, F) Cell uptake behavior of LU NPs released from LU@gel observed by fluorescence inverted microscope and flow cytometry. Scale bar: 20 μm. (G) The impact of intracranial in situ tamponade with LU@gel on brain tissue. Scale bar: 1.0 mm.

We mixed LU NPs with PVA and subjected them to repeated freeze-thawing to create a PVA hydrogel loaded with LU NPs. SEM revealed that all hydrogels were macroporous, featuring large and interconnected pores. The average pore size ranged from approximately 105 μm for 2 % PVA to 36.5 μm for 8 % PVA. The addition of LU NPs increased the pore size of the LU@gel (Fig. 5A). In rheological tests, within the frequency range of 0.1–10Hz, G′ was significantly greater than G″, indicating the formation of the hydrogel (Fig. 5B). We found that adding LU NPs to 8 % PVA resulted in an LU@gel with a G′ of about 2000Pa, which is close to the Young's modulus of brain tissue, making it suitable for further use. Surface elemental composition analysis via XPS revealed that, unlike PVA, the surface C and O elemental ratios of LU, LU NPs, and LU@gel are similar. This indicates that LU NPs are likely predominantly attached to the surface of PVA, providing a structural foundation for LU release (Fig. 5C). We then measured the release behavior of the LU@gel. The LU@gel was prepared and placed in a PBS solution under stirring, and the release of Luteolin was quantified by measuring the absorbance at 353 nm using a UV spectrophotometer. As illustrated in Fig. 5D, LU@gel demonstrated sustained LU release over a 24-day period. Effective drug release characteristics were maintained during the initial 12 days, whereas a marked reduction in release efficiency was observed between days 12 and 24. By encapsulating fluorescent dye Coumarin 6 into LU NPs, we further examined the cellular uptake behavior of LU NPs in the LU@gel. As shown in Fig. 5E and F and S3, the LU NPs in the LU@gel were significantly released within 6h and taken up by GL261 cells. To further investigate the safety of in situ packing with LU@gel, we chose tumor-bearing C57BL/6J mice, removed the tumor, packed the area with LU@gel gel, and sacrificed the mice three days later to collect brain tissue for HE staining. As shown in Fig. 5G, the brain tissue of mice in the blank group exhibited extensive bleeding, while the amount of bleeding in the LU@gel group was reduced, and no significant edema or cell death was observed in brain tissue cells in contact with the LU@gel. This indicates that the LU@gel has good biocompatibility and can be further used in in vivo experiments.

### 3.6 Characterization of LU@gel for the treatment of GBM In Vivo

**Fig. 6:**
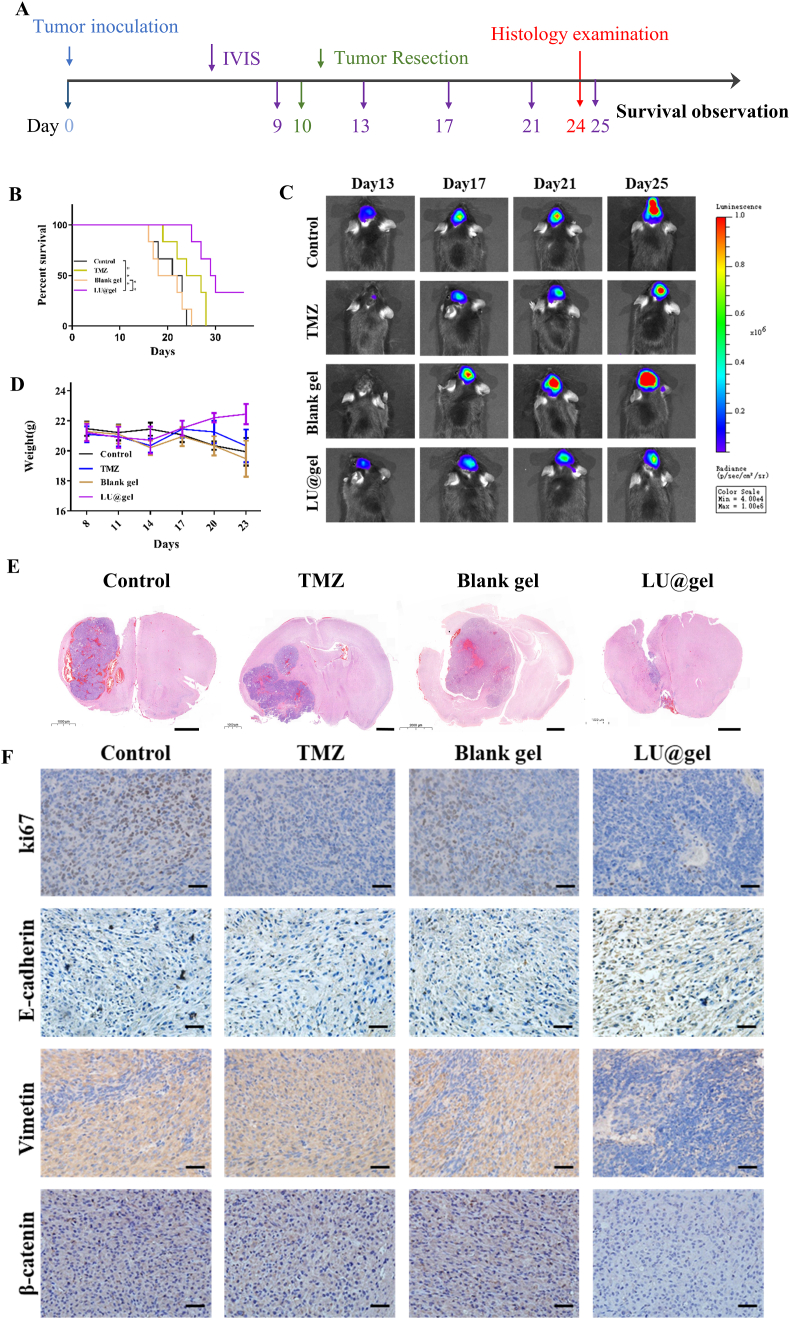
**Characterization of LU@gel for the Treatment of GBM In Vivo.** (A) Schematic of the experimental timeline. (B) Kaplan–Meier curves showing mouse survival rates. (n = 6) ∗∗P < 0.01, ∗∗∗P < 0.001. (C) Representative IVIS images of tumor from mice in Control, TMZ, Blank gel and LU@gel group. (n = 5). (D) Changes in mouse body weight. (n = 7). (E) Representative HE images of mouse brain sections from mice in Control, TMZ, Blank gel and LU@gel group. Scale bar: 1.0 mm. (F) IHC staining images for Ki67, E-cadherin, Vimetin, and β-catenin. Scale bar: 20 μm.

We assessed the therapeutic efficacy of LU@gel in treating glioblastoma in vivo, as illustrated in Fig. 6A. Using IVIS imaging, we observed that the tumor recurrence rate in the LU@gel group was significantly lower compared to the Blank gel and TMZ groups (Fig. 6C and S4). Survival analysis further demonstrated that mice treated with LU@gel had a markedly improved survival rate compared to those receiving Blank gel (P < 0.05). The survival rate in the LU@gel group was also significantly higher than that in the TMZ group, suggesting substantial clinical benefits of LU@gel treatment (Fig. 6B). Monitoring of body weight during the acute postoperative period revealed no significant changes, indicating that the hydrogel did not exhibit noticeable toxicity when used as an in situ filler in the brain (Fig. 6D and S5). Upon further comparison of the tumor size in the brain tissues taken on the 24th day post-surgery, we also found that the recurrent tumors in the LU group were significantly smaller than those in the other groups, which further demonstrates the remarkable efficacy of LU treatment (Fig. 6E and S6). Immunohistochemical analysis of brain tissues showed a significant reduction in the number of proliferative tumor cells, as indicated by Ki67 staining, in the LU@gel group compared to the PBS and Blank gel groups (Fig. 6F and S7). Consistent with our in vitro findings (Fig. 3G), LU@gel treatment led to a notable increase in E-cadherin expression and a decrease in the levels of Vimentin and β-Catenin in the tumors (Fig. 6F). These results suggest that LU@gel effectively inhibits GBM progression by targeting the β-Catenin/EMT signaling pathway.

## 4 Conclusion

In summary, we have demonstrated that LU@gel exhibits potent anti-GBM effects, primarily attributed to the inhibition of β-catenin-mediated cell proliferation and EMT. Given its remarkable anti-GBM efficacy and superior safety profile, LU@gel holds great potential for translation into clinical applications to improve the clinical management of GBM.

## CRediT authorship contribution statement

**Long Zhou:** Writing – original draft, Validation, Software, Methodology, Investigation, Data curation, Conceptualization. **Qingyu Zhao:** Visualization, Validation, Software, Investigation, Formal analysis. **Lijuan Gu:** Supervision, Software, Project administration, Formal analysis. **Renfu Tian:** Data curation, Conceptualization. **Yong Li:** Writing – review & editing, Writing – original draft, Conceptualization. **Xiaoxing Xiong:** Writing – review & editing, Resources, Funding acquisition, Conceptualization.

## Ethics approval and consent to participate

The Committee of Animal Care and Use of Renmin Hospital of Wuhan University approved all experiments with animals in this study.

## Consent for publication

Not applicable.

## Availability of data and materials

The data used to support the findings of this study are available from the corresponding author upon request.

## Declaration of competing interest

The authors declare that they have no known competing financial interests or personal relationships that could have appeared to influence the work reported in this paper.

## Data Availability

Data will be made available on request.
