# Supplementary material for: Application of PVA hydrogel loaded with luteolin nanoparticles in anti EMT treatment after GBM
Source: Mater Today Bio. 2025 Jun 7;33:101956. doi: 10.1016/j.mtbio.2025.101956 (PMC12213306; doi:10.1016/j.mtbio.2025.101956)
Supplement: Multimedia component 1 [file mmc1.pdf]

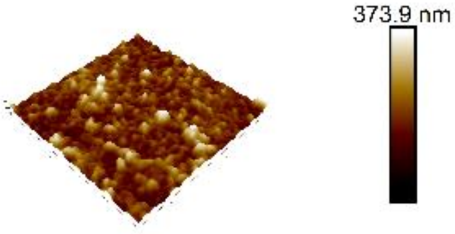

Figure S1. Representative 3D AFM image of LU NPs.

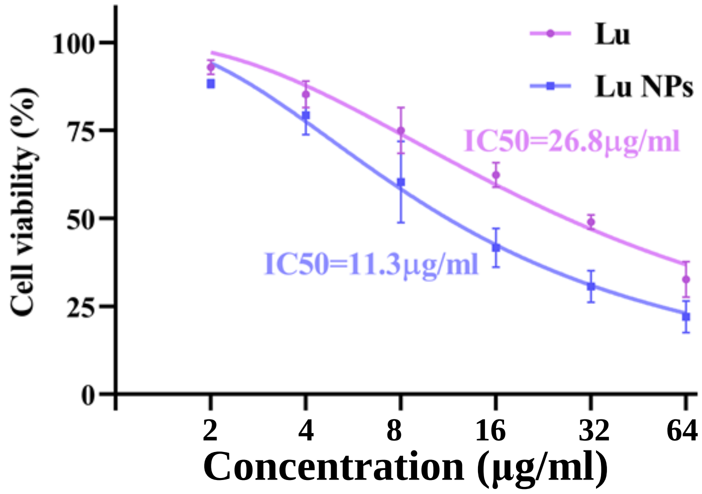

Figure S2. Cell viability measured by CCK8 assay after LU NPs treatment with various concentrations (0, 2 ,4 ,8 ,16 ,32, 64 ug/ml). (n=3).

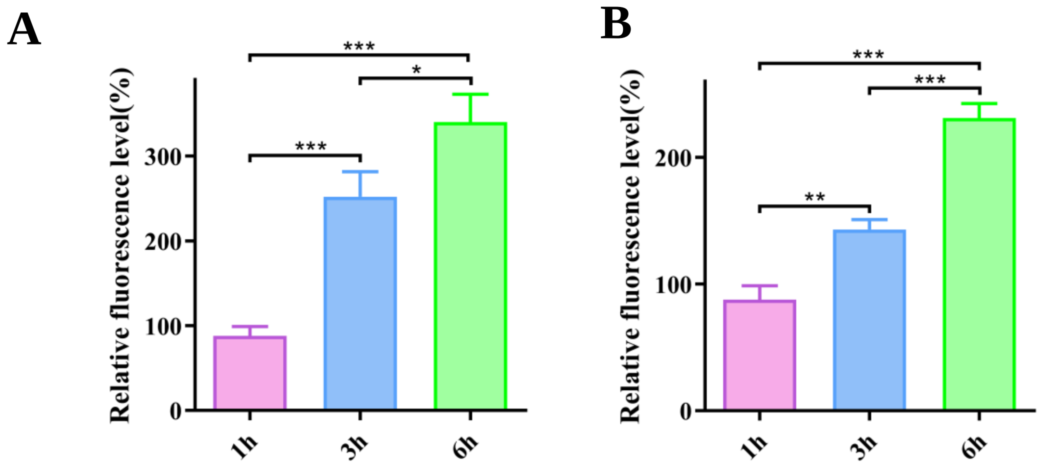

Figure S3.Analysis of cell uptake behavior of LU NPs released from LU@gel observed by fluorescence inverted microscope (A) and flow cytometry (B). *P < 0.05, **P < 0.01, ***P < 0.001, (n=3).

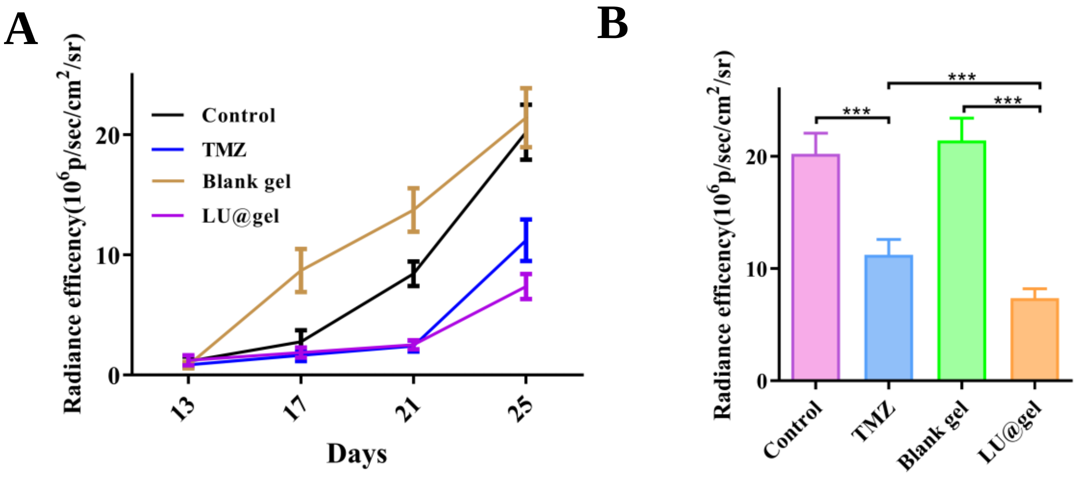

Figure S4. IVIS of tumor from mice (A) and Analysis of IVIS (B) in day 25 in Control, TMZ, Blank gel and LU@gel group. *P < 0.05, **P < 0.01, ***P < 0.001, (n=5).

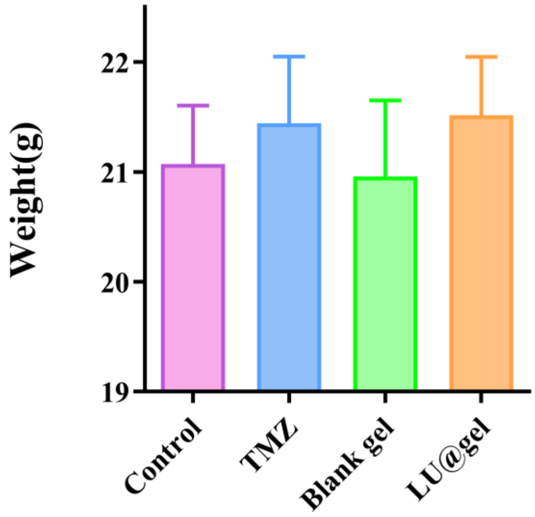

Figure S5. Analysis of mouse body weight in Control, TMZ, Blank gel and LU@gel group in day 17. (n = 7).

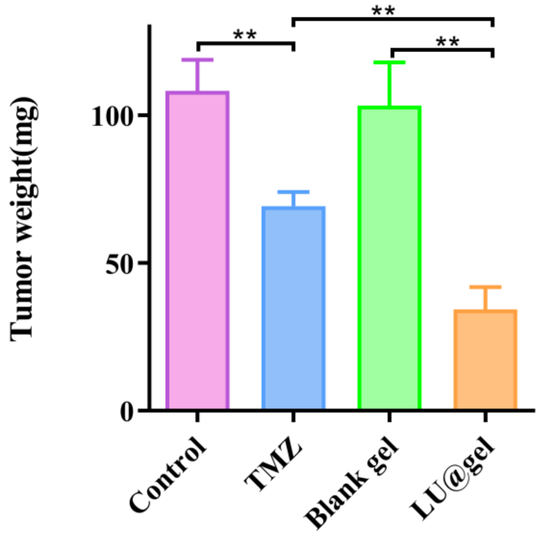

Figure S6. Analysis of mouse tumor weight in Control, TMZ, Blank gel and LU@gel group. *P < 0.05, **P < 0.01, ***P < 0.001, (n = 3).

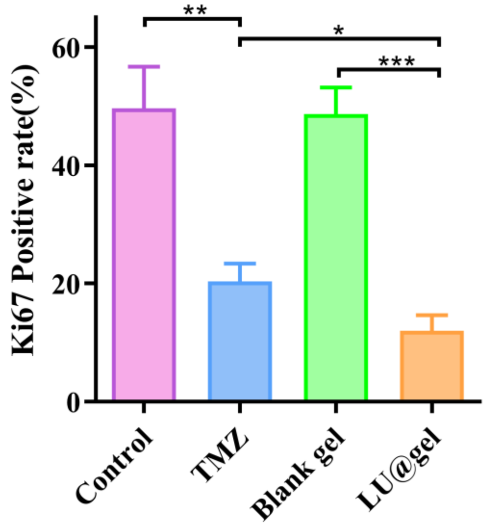

Figure S7. Ki67 positive rate in mouse tumor in Control, TMZ, Blank gel and LU@gel group. *P < 0.05, **P < 0.01, ***P < 0.001, (n = 3).
