# Supplementary figures and images for: Application of PVA hydrogel loaded with luteolin nanoparticles in anti EMT treatment after GBM
Source: Mater Today Bio. 2025 Jun 7;33:101956. doi: 10.1016/j.mtbio.2025.101956 (PMC12213306; doi:10.1016/j.mtbio.2025.101956)

Marker: Servicebio Prestained Protein Marker X(10-180 kDa)

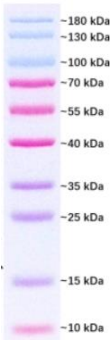

Figure 2F

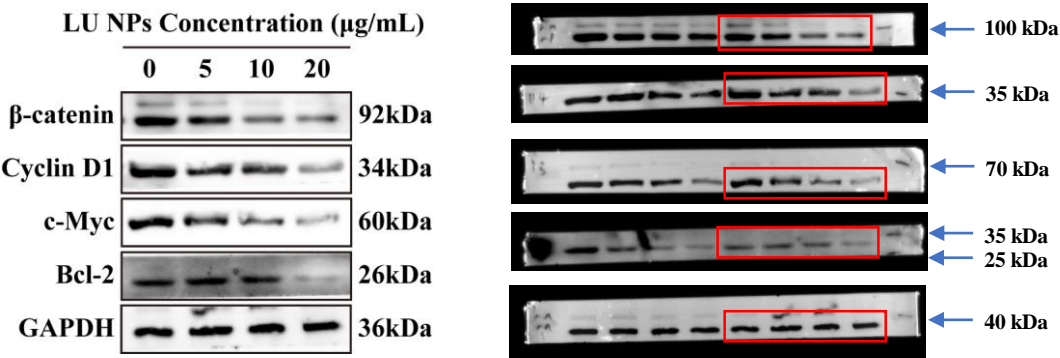

Figure 3G

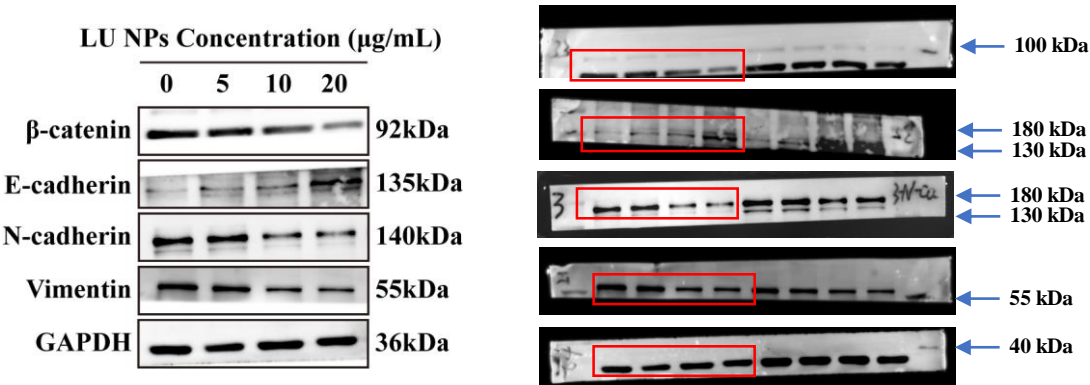

Figure 4A

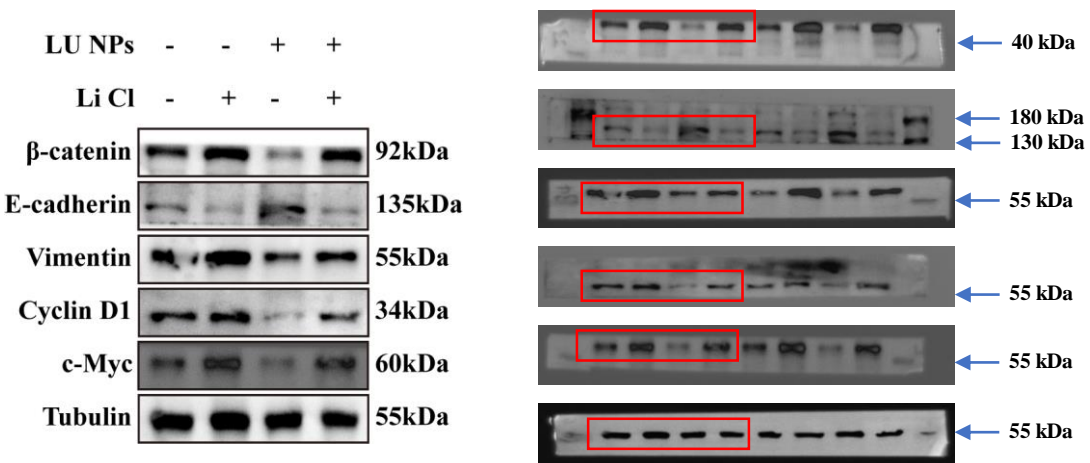

Supplement: Multimedia component 2 [file mmc2.pdf]
